# Multi-slice ptychographic tomography

**DOI:** 10.1038/s41598-018-20530-x

**Published:** 2018-02-01

**Authors:** Peng Li, Andrew Maiden

**Affiliations:** Department of Electronic and Electrical Engineering, University of Sheffield, Sheffield, S1 3JD UK

## Abstract

Ptychography is a form of Coherent Diffractive Imaging, where diffraction patterns are processed by iterative algorithms to recover an image of a specimen. Although mostly applied in two dimensions, ptychography can be extended to produce three dimensional images in two ways: via multi-slice ptychography or ptychographic tomography. Ptychographic tomography relies on 2D ptychography to supply projections to conventional tomographic algorithms, whilst multi-slice ptychography uses the redundancy in ptychographic data to split the reconstruction into a series of axial slices. Whilst multi-slice ptychography can handle multiple-scattering thick specimens and has a much smaller data requirement than ptychographic tomography, its depth resolution is relatively poor. Here we propose an imaging modality that combines the benefits of the two approaches, enabling isotropic 3D resolution imaging of thick specimens with a small number of angular measurements. Optical experiments validate our proposed method.

## Introduction

Ptychography is most often implemented as a two-dimensional technique, producing phase images that represent a projection of the optical path length through a sample^1,2^. This requires that the sample is optically thin, such that it falls within the multiplicative approximation where the wavefront that passes through a sample can be accurately modelled as the multiplication of the incident illumination function and a sample transmission function^3–5^. The thickness limit for this approximation has been theoretically studied via different means^1,2^, and refined via numerical calculations to give the following relation^6^

1 $$T\le \frac{5.2{\rm{\Delta }}{r}^{2}}{\lambda },$$

where Δ*r* is the image resolution and *λ* is the wavelength of the illumination. This equates to a Fresnel number no smaller than 0.2.

**Figure 1:**
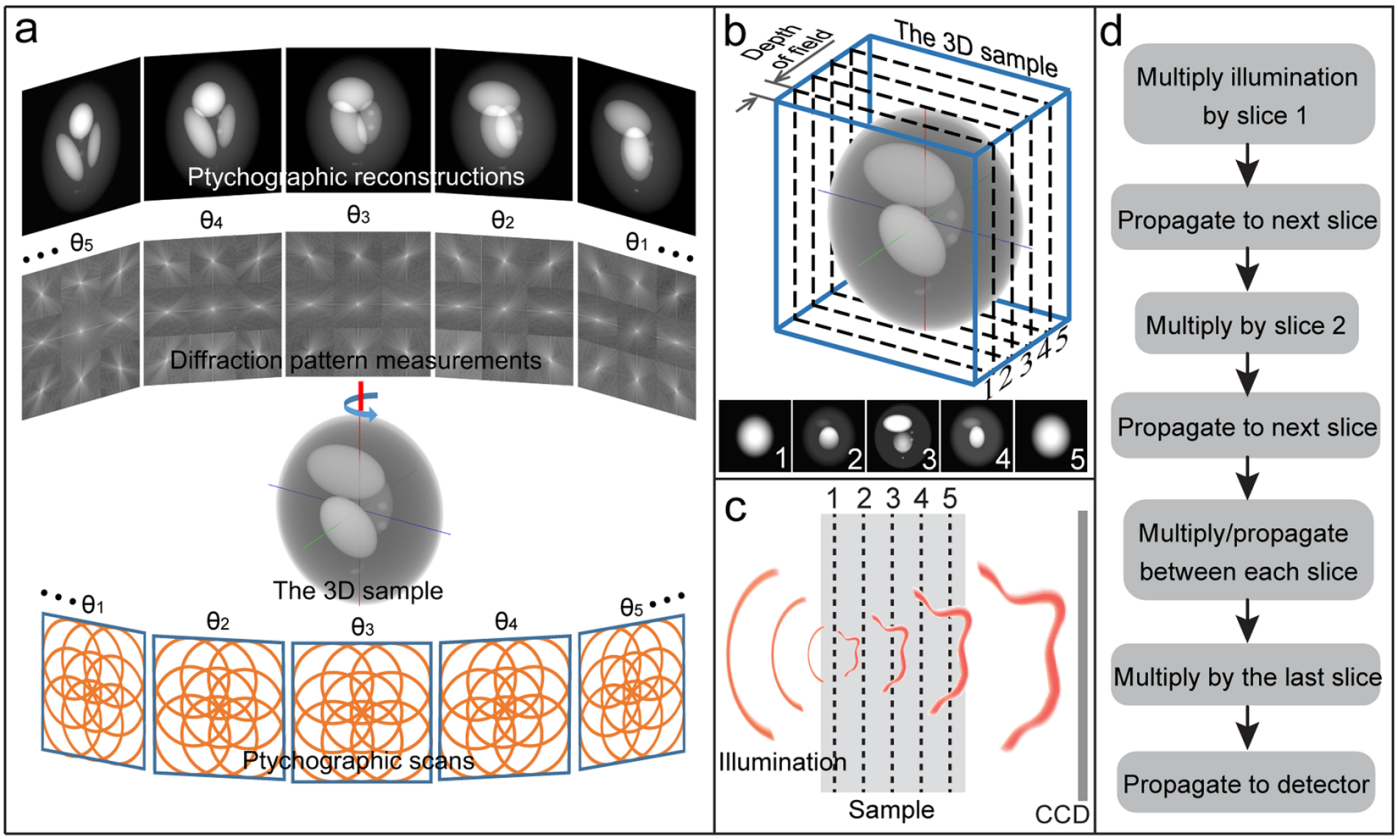
3D ptychography. (**a**) Schematic diagram of ptychographic tomography. (**b**) Sectioning a thick sample into a set of thin slices when the sample thickness exceeds the depth of field of single-slice ptychography. (**c**) Schematic diagram of the illumination propagating through a sliced thick sample and reaching the detector. (**d**) Flowchart of the multi-slice calculation model.

Although two-dimensional ptychography can accommodate a degree of multiple scattering^1^, once Eq. (1) is violated conventional ptychographic algorithms fail, because they fail to take account of the propagation happening within the sample. On the other hand, when a sample is thin enough to satisfy Eq. (1), ptychographic reconstructions can be combined with tomography to produce high resolution 3D images; this combination is termed ptychographic tomography and is implemented as shown in Fig. 1a. To obtain 3D images from this setup, the sample is rotated through 180 degrees in small steps, and for each angular orientation a single-slice ptychographic experiment is carried out to obtain a projection of the sample. Once projections have been reconstructed from each angle, they are assembled together using standard tomographic procedures, for example the filtered back projection algorithm (FBP)^7^, to give the final 3D image.

The popularity and success of this technique in the x-ray regime^8–10^ is attributable to several advantages: 1. an imaging lens is not necessary for ptychography, leading to a very simple experimental setup and high image resolutions (limited only by the detector collection angle)^1,11^; 2. a large volume can be imaged, thanks to the extended field of view allowed by the sample translations in ptychography^4,12^; 3. the reconstructed phase image not only has good contrast, but also allows quantitative analysis of the sample^13,14^. However, the drawback of this technique is that data collection is very time-consuming. Ptychography needs dense translational scans because of the overlap constraint^15^ and tomography needs dense angular scans because of the Crowther Limit^16^. Together these factors result in experiments of many hours^8,10^. Inevitably during this long scan process, radiation damage and sample drift are introduced that further limit the appeal of the method.

When the sample thickness exceeds the limit of the multiplicative approximation, a computational model called the multi-slice calculation^17^ can be adopted to calculate the exit wave function of the sample. This model computationally sections the thick sample into a set of thin slices, each of which falls within the multiplicative approximation, as shown in Fig. 1b. The experimental process of one scan position then follows the diagram in Fig. 1c. Using this process, the wave in the detector plane can be calculated via the calculation flowchart given in Fig. 1d: the illumination function is multiplied by the first slice, then propagated to and multiplied by the second slice, then propagated to and multiplied by the third, and so on until the last slice is reached to give the final exit wave, and propagating this exit wave to the detector gives the calculated wave in the detector plane. For the image reconstruction, the calculated wave is first revised by replacing its moduli with the square root of the measured intensity and then the whole calculation flowchart is reversed. Each of the thin slices in the multi-slice model can be reconstructed using the update functions from the conventional single-slice ptychographic algorithm^18^, hence providing a 3D image of the sample. Full details of the algorithm can be found in the original paper^19^.

For multi-slice ptychography, the data collection process is exactly the same as single-slice ptychography. This is advantageous because no additional sample rotation is needed to provide 3D information. It also extends the thickness limit that ptychography can deal with. However, its depth resolution is rather low compared to its lateral resolution. Despite proof-of-principle results with x-rays^20^ and electrons^21^, multi-slice ptychography is easiest to implement in the visible light regime, thanks to the stronger interaction with matter and the availability of high NA optics at these longer wavelengths^19,22^. These two factors aid the clean separation of slices during a multi-slice reconstruction, which relies on considerable difference between the illuminations of each of the slices.

Given the complementary benefits of multi-slice ptychography and ptychographic tomography, it is intuitive to attempt a combination of the two techniques. In this paper, we propose using the limited 3D information from multi-slice ptychography to reduce the number of angular measurements (and scan time) needed for ptychographic tomography, whilst increasing the sample thickness that tomography can accommodate and retaining its high isotropic 3D resolution. We experimentally demonstrate the ability of this combination to extend the sample thickness and reduce the angular measurements. Hereinafter, this new technique will be referred to as multi-slice ptychographic tomography (MSPT) and the conventional ptychographic tomography as single-slice ptychographic tomography (SSPT).

## Results

**Figure 2:**
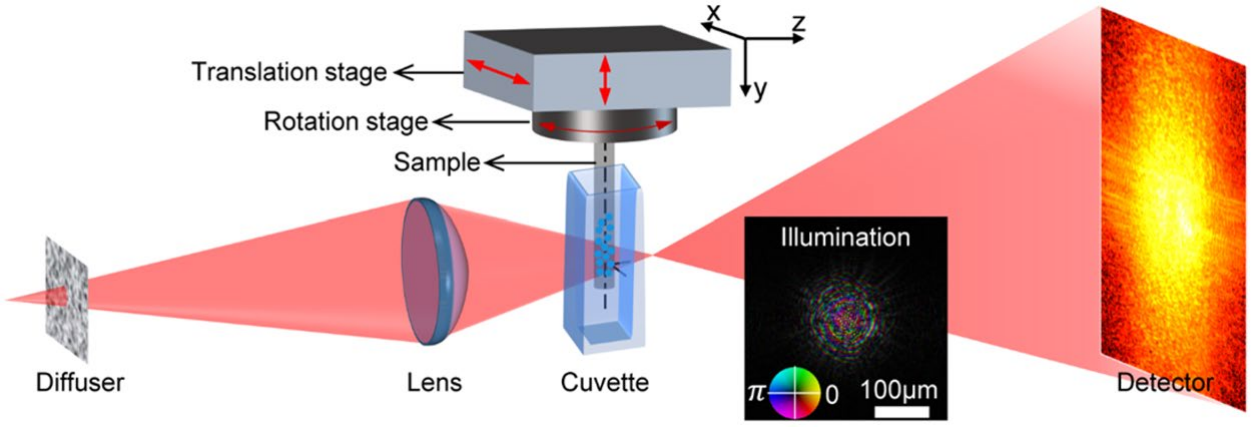
The experimental setup of MSPT. For the illumination, the color wheel depiction shows phase variations as changes in hue and modulus variations as changes in brightness.

Figure 2 shows the setup of the optical experiment we implemented to investigate the benefits of MSPT. A diffuser and a lens were used to form a localised illumination (see the inset in Fig. 2) onto the sample, which constituted a glass tube filled with glass beads (a few of which were hollow) immersed in index-matching liquid to reduce scattering strength. The sample was rotated to 90 evenly spaced angles spanning 180°, and for each angle a ptychographic scan with 3 × 15 raster positions was performed.

**Figure 3:**
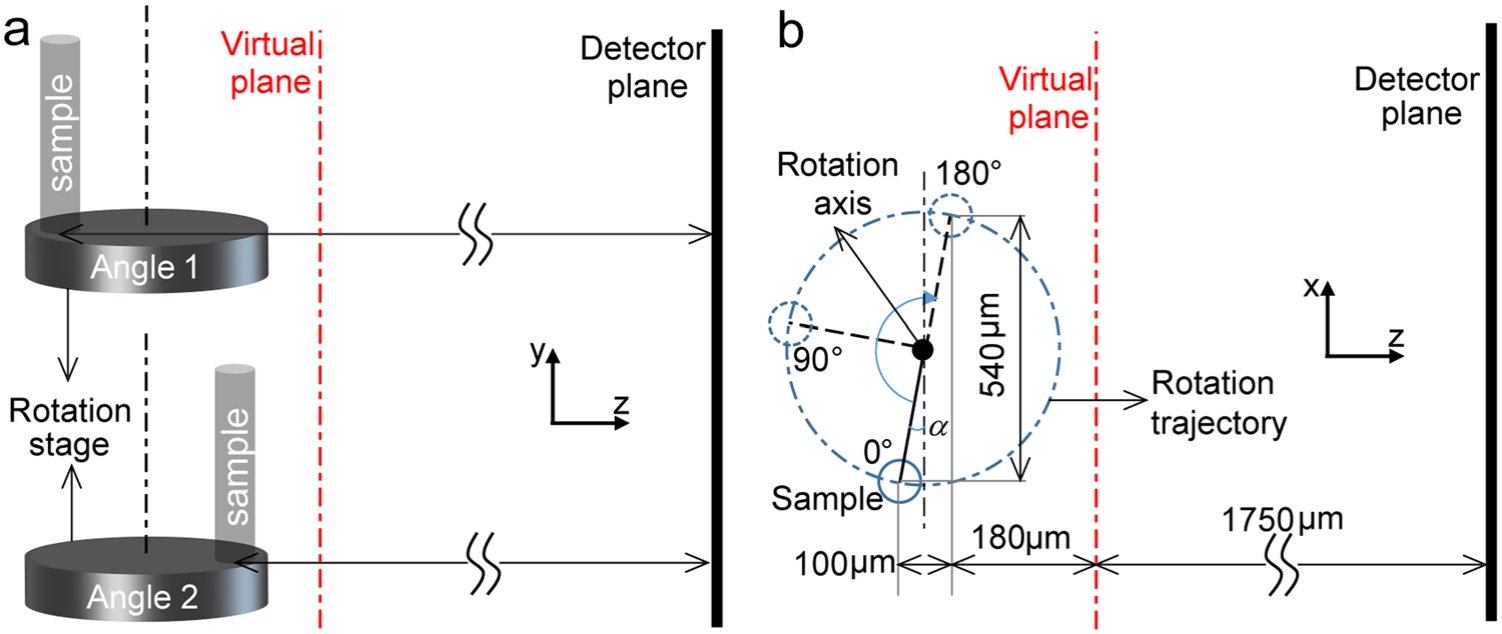
Schematic diagram of the rotation geometry of the sample. (**a**) Side view. (**b**) Top view.

Because of inevitable mounting errors, the y-axis of the tube was not perfectly aligned with the rotation axis, as shown in Fig. 3a. This resulted in the distance from the sample to the camera changing as the sample was rotated. When a Fresnel propagator (the Fourier representation of the Fresnel propagation) or a Fourier far-field (Fraunhofer) propagator^23^ is used for the propagation between the sample and the detector, this causes the pixel size to change between the reconstructed images^24^. To ensure a fixed pixel size for the following tomographic reconstruction, a ‘virtual plane’ at a position 17.5 mm from the detector was included in the reconstruction process (see Fig. 3b and details in the Methods section).

### SSPT reconstructions using all angles

The pixel size of the ptychographic reconstructions was 733 nm as a result of the virtual plane’s position selection. Since the diffraction pattern fills the whole detector, as shown in Fig. 2, we can approximate the image resolution as twice the pixel size, which gives us 1.47 µm. From this, Eq. (1) suggests the depth of field for this setup as at most 26.9 µm (with consideration of refractive index change introduced by the index matching fluid), which is about 18 times the lateral resolution. Since the sample thickness (set by the glass tube’s 90 µm outer diameter) far exceeds this figure, a multi-slice strategy is necessary to obtain accurate ptychographic reconstructions. Despite this, we first carried out single-slice ptychographic reconstructions for each sample rotation angle to implement SSPT. Although our analysis suggests this will produce a poor final 3D image, it will nevertheless serve as a baseline for comparison with the MSPT 3D image, and it will also provide a useful initial seed for the multi-slice ptychographic reconstructions.

**Figure 4:**
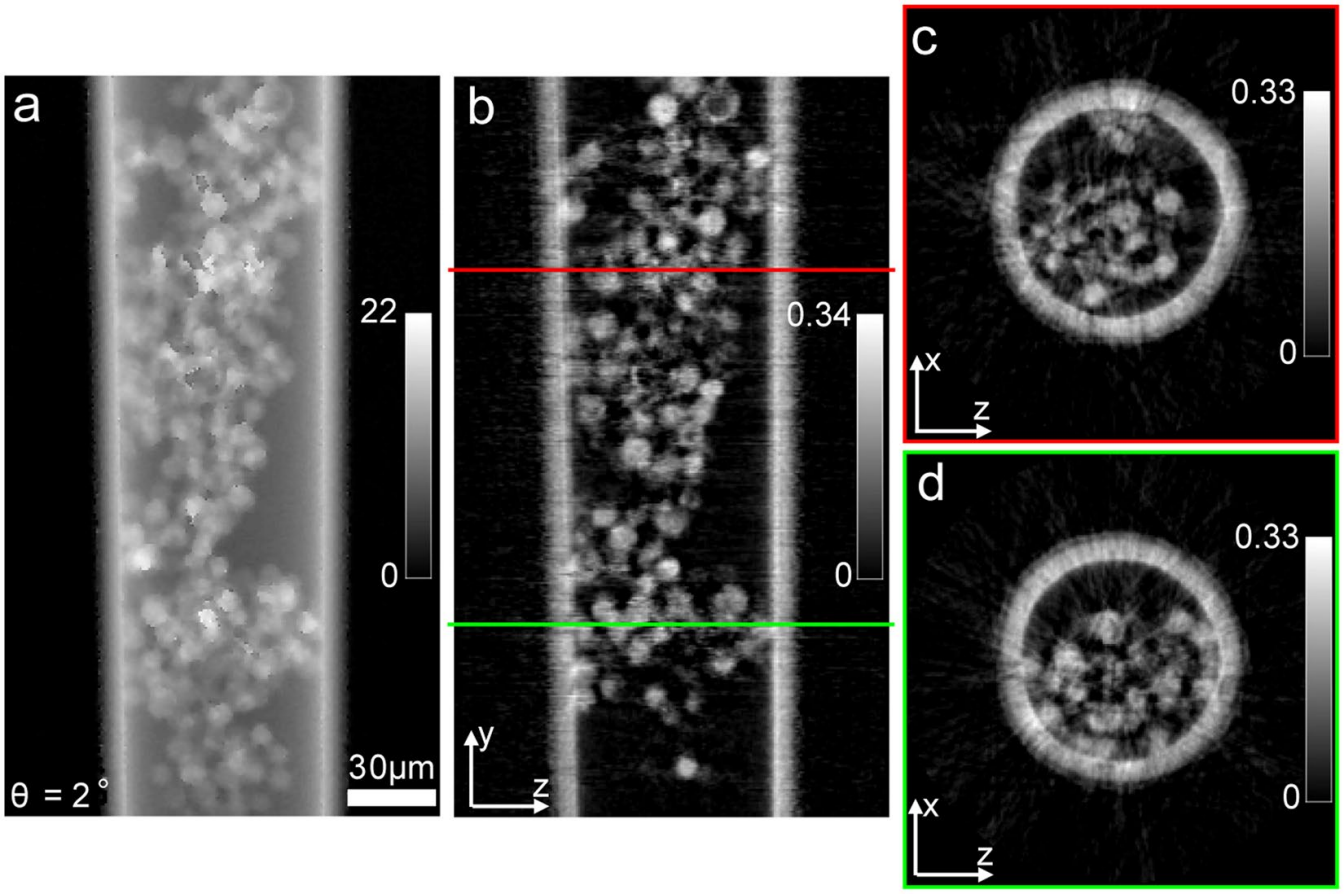
Reconstructions of SSPT. (**a**) The phase part of the single-slice ptychographic reconstruction of the sample at angle of 2°. (**b**) The y-z central cross-section of the 3D sample reconstruction. (**c**) The x-z cross-section at the position indicated by the red line in (**b**). (**d**) The x-z cross-section at the position indicated by the green line in (**b**).

For the single-slice reconstructions, we used the regularized PIE (rPIE) algorithm described in^25^. An example of the resulting (unwrapped^26^) phase image of the sample is shown in Fig. 4a. The reconstruction is especially poor where the beads are dense, because of the strong multiple scattering effects in these regions; this presents problems for phase unwrapping, in turn resulting in the patchwork of artefacts evident in the Figure.

Having obtained via ptychography (somewhat poor) projections of the sample at each rotation angle, further pre-processing is needed prior to tomographic reconstruction to compensate for ambiguities inherent to ptychographic images, which include a constant phase offset, a linear phase ramp and a global translation^27,28^. The approach described in reference^27^ was used for the removal of the constant phase offset and the linear phase ramp, making use of the fact that free space should cause zero phase change to the illumination wavefield. The global translation was corrected by aligning the phase reconstructions. Here we used the cross-correlation registration of neighboring projections^29^ to implement the alignment. To avoid strong artefacts caused by the poor phase unwrapping, we used a modified FBP algorithm to perform the tomographic reconstruction, which replaces the unwrapped phase projections with the derivative of the phase, as described in refs^7,27^. The phase derivative was calculated by:

2 $$\frac{\partial \varphi (x,y,\theta )}{\partial x}\approx \frac{1}{h}{\rm{\arg }}\{\exp [i\varphi (x+\frac{h}{2},y,\theta )]\,\exp \,[-i\varphi (x-\frac{h}{2},y,\theta )]\},$$

where $\varphi (x,y,\theta )$ is the unwrapped phase reconstruction at rotation angle *θ*, *h* is the pixel step for the phase shift along the x-axis and the ${\rm{\arg }}\{\cdot \}$ operation represents the extraction of the phase part. Here we used a pixel step of 1 for the derivative calculation and the subpixel shifts were implemented using the method described in reference^25^. Example cross-sections of the resulting 3D reconstruction are shown in Fig. 4b,c and d. As expected, depth of field limitations have resulted in artefacts. However, the reconstruction is good enough to reveal some 3D morphological features of the sample.

### MSPT reconstructions using all angles

**Figure 5:**
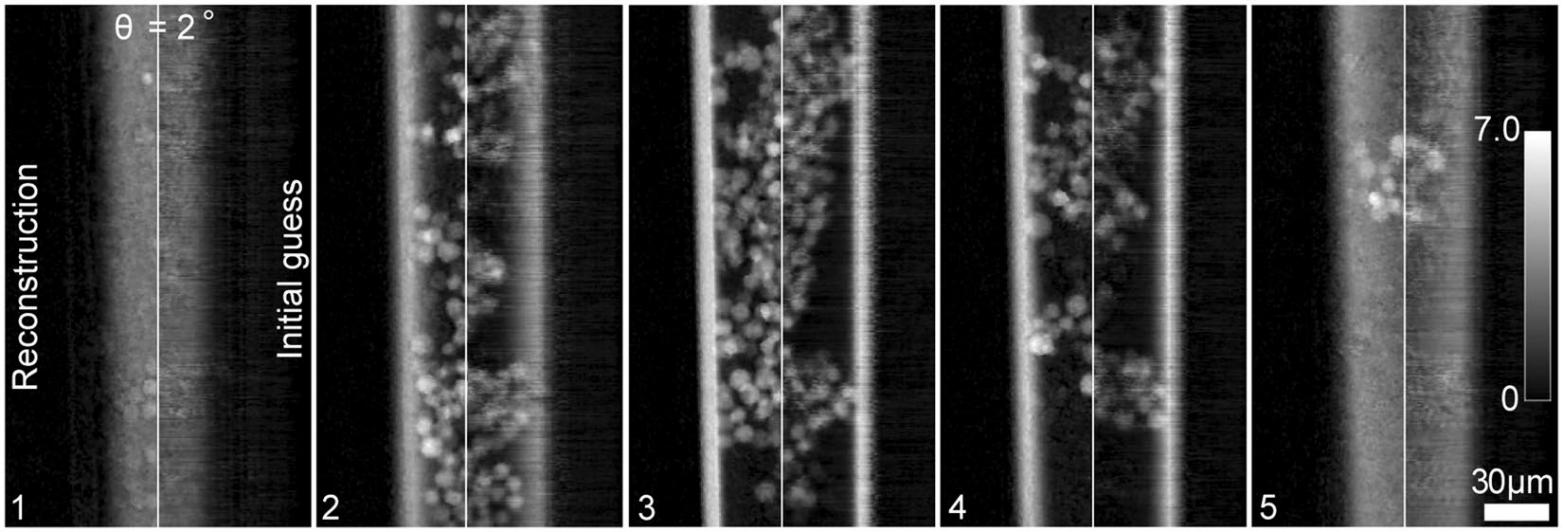
The comparison between the initial guesses (the right hand side in each panel) and the reconstructions (the left hand side) from the multi-slice ptychography.

For the multi-slice reconstruction, 5 slices with a spacing of 18 µm are enough to cover the sample thickness, with each slice well within the limit set out by Eq. 1. To use the SSPT 3D image (Fig. 4) as a seed for the multi-slice process we split it into 5 slices – example slices from the 2° orientation are shown to the right hand side of the panels in Fig. 5. The initial modulus guesses for the slices were arrays of 1 s. We used the multi-slice strategy described in^15^ coupled with the update functions from the regularized PIE algorithm^21^ to carry out the multi-slice reconstructions. The improved images for the 5 slices at 2° are shown to the left hand side of the panels in Fig. 5.

**Figure 6:**
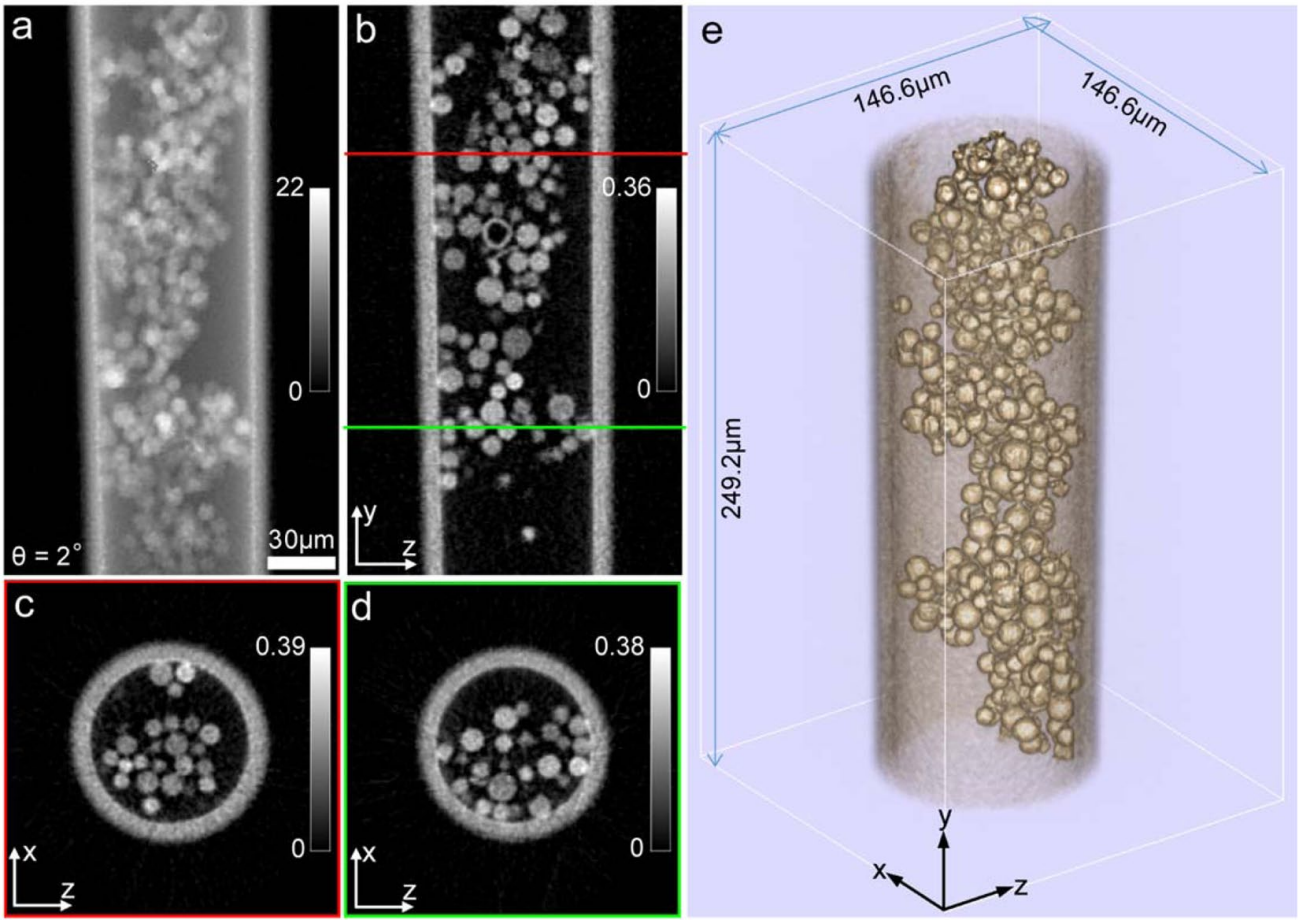
Reconstructions of MSPT. (**a**) The phase projection of the sample from the multi-slice ptychographic reconstruction at angle of 2°. (**b**) The y-z central cross-section of the 3D sample reconstruction. (**c**) The x-z cross-section at the position indicated by the red line in (**b**). (**d**) The x-z cross-section at the position indicated by the green line in (**b**). (**e**) 3D rendering of the sample reconstruction.

Having obtained the multi-slice reconstructions for all the angles, the phase projections of the sample at each angle were calculated by pixel-wise addition of the phases of the corresponding 5 slices^6^. Figure 6a shows the phase projection at angle 2°, which is significantly better than its single-slice reconstruction (see Fig. 4a). The improvement is attributed to the fact that the multi-slice ptychographic reconstruction accounts for propagation and multiple scattering effects inside the sample. Because the unwrapped phase reconstructions here were artefact-free, we adopted the mass-based method^30^ to implement the alignment because of its superiority to the cross-correlation method. (The ‘mass’ is defined to be the integral of the phase projection). Since the field of view of ptychography covers the whole sample’s horizontal width but not its vertical height, the alignment methods for the x- and y- axes were different: the alignment along the x-axis was obtained via the centre of mass approach^27,30^, and the alignment along the y-axis was achieved by maximizing the correlation of sample mass fluctuations^27^. For the tomographic reconstruction, we used the same modified FBP algorithm as described above. Example cross-sections of the resulting 3D reconstruction are shown in Fig. 6b,c and d. Compared to the results shown in Fig. 4, artefacts are considerably reduced and the profiles of the beads and the tube correspond much more closely with the real sample. Moreover, from the y-z central cross-section (Fig. 6b), we can see the interior of one of the hollow beads, which is not visually discernible in Fig. 4b. A 3D rendering of the sample reconstruction is shown in Fig. 6e, which gives a clear view of the connections of the beads inside the tube.

### MSPT reconstructions using limited angles

**Figure 7:**
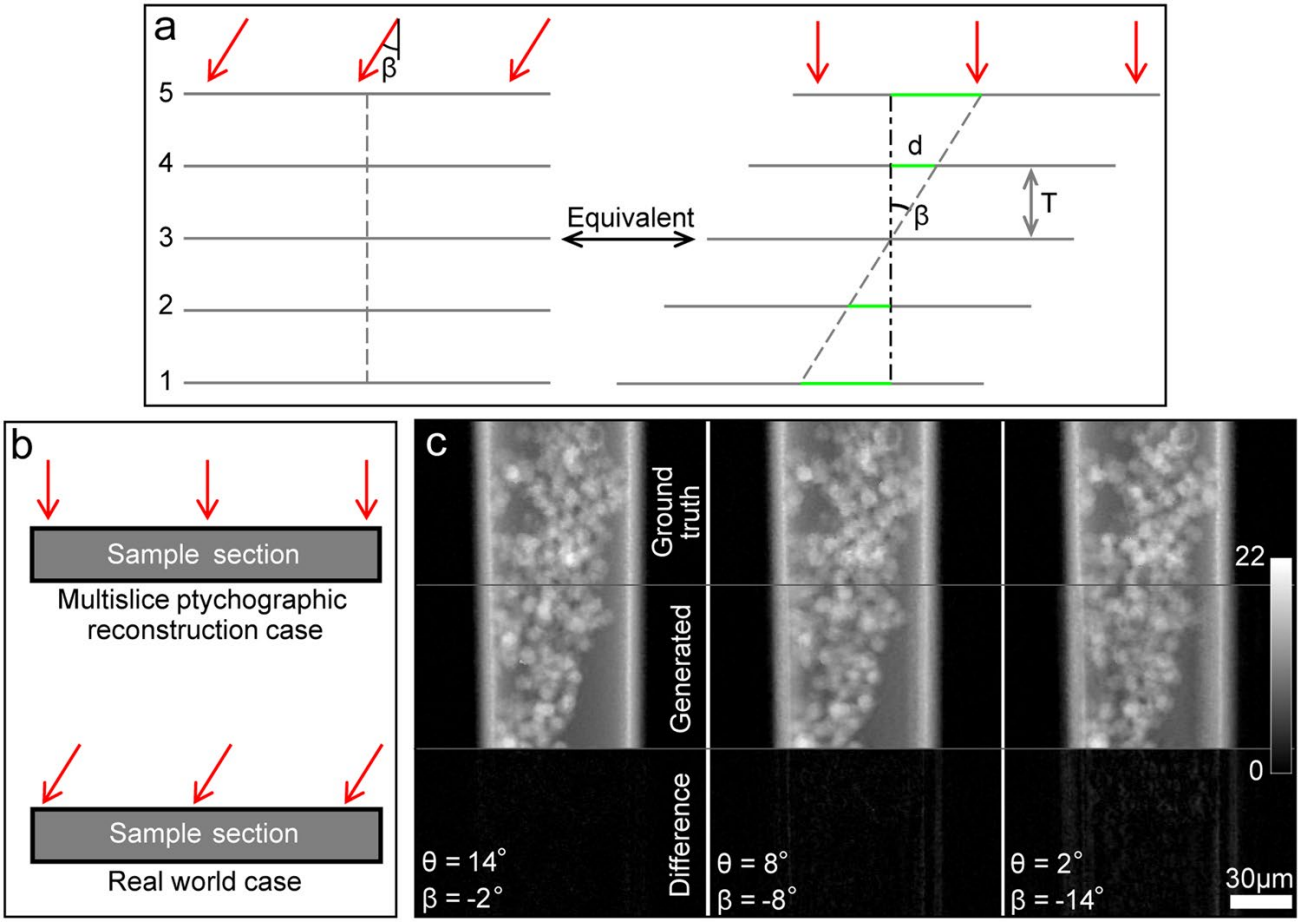
Generating projections from multi-slice reconstructions. (**a**) Schematic diagram showing the equivalence between the projection of a sample with discrete slices under a tilted view angle and its orthogonal projection by offsetting the slices relative to each other when the slices are infinitesimally thin. (**b**) Schematic diagram showing the difference between the orthogonal projection produced by multi-slice reconstruction and the ground truth projection under a tilted view angle for a sample section with certain thickness. (**c**) The phase projections of the sample generated from the multi-slice reconstructions at the angle of 16° by tilting the view angle by −2°, −8° and −14°.

The number of angular projections used by the FBP algorithm cannot be reduced without affecting image quality (unless compressive sensing is applied^31^). With that in mind, the problem comes down to how to use the multi-slice reconstructions from one particular sample orientation to generate projections for a range of angles for input to the FBP step. For a sample with discrete slices of infinitesimal thickness, the projection corresponding to a particular tilted view angle can be formed by laterally offsetting the slices relatively to each other by a certain amount, as depicted in Fig. 7a. The view angle *β* and the distance $T$ between two slices determines their relative offset *d*, since

3 $$d=T\,\tan \,\beta .$$

This idea can be adopted for the multi-slice reconstructions to generate sample projections. However, each slice from the multi-slice reconstruction represents the orthogonal projection of the corresponding sample section, which is not infinitesimally thin and so is not identical to the true projection of a tilted view angle, as Fig. 7b shows. It is not difficult to see that the difference between these two projections depends on the section thickness and the view angle. For a fixed view angle, the smaller the section thickness, the smaller the difference would be. On the other hand, for a fixed section thickness, as in the multi-slice reconstruction case here, the smaller the view angle, the smaller the difference would be. Figure 7c shows three phase projections at the angles of 14°, 8° and 2°. The ground truth projections were calculated from the multi-slice ptychographic reconstructions of the respective angles. The approximated projections were calculated via the offset strategy using exclusively the multi-slice reconstruction from the 16° rotation angle. The distance between two adjacent slices is the section thickness we used for the multi-slice reconstruction, i.e. 18 µm. The offsets for all the slices were calculated using the middle slice as the reference plane, which means the distance $T$ was calculated relative to this plane. The offsets calculated from Eq. (3) were converted to pixel offsets by dividing them by the pixel size. Subpixel shifts were implemented using the method described in reference^32^. As Fig. 7c shows, when the tilted view angle *β* is −2° (minus sign here means anti-clockwise), the difference between the generated projection and the ground truth projection is very small. However, when the tilt angle is increased, the difference increases noticeably.

**Figure 8:**
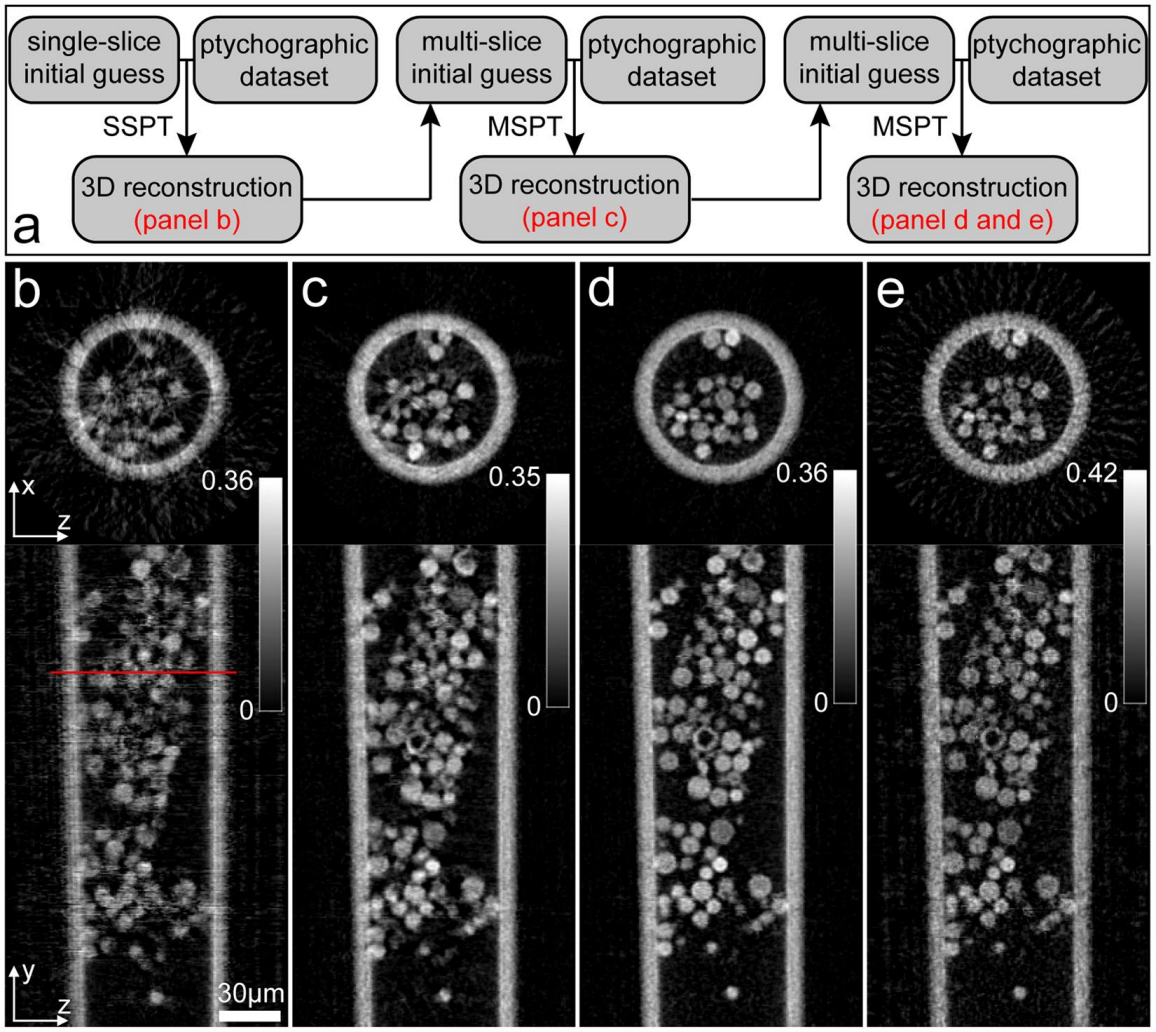
The reconstructions of multi-slice ptychographic tomography using 30 angular measurements. (**a**) The reconstruction pipeline. (**b**) Cross-sections of the tomographic reconstruction using the selected 30 reconstructions from the single-slice ptychography. (**c**) Cross-sections of the tomographic reconstruction using the generated 90 projections from the first multi-slice ptychographic reconstructions. (**d**) Cross-sections of the tomographic reconstruction using the generated 90 projections from the second multi-slice ptychographic reconstructions. (**e**) Cross-sections of the tomographic reconstruction using the 30 orthogonal projections from the second multi-slice ptychographic reconstructions.

The reasoning above suggests a reasonable angular range of projections can be recovered from a single multi-slice reconstruction. To test this hypothesis, we extracted from our original 90-angle dataset 30 angles between 4° and 178° spaced 6° apart. A series of reconstructions were carried out using the ptychographic datasets from this selection of 30 angles. The reconstruction pipeline is shown in Fig. 8a. SSPT was first performed to give a rather poor 3D reconstruction of the sample, as shown in Fig. 8b. Clearly the result is inferior to that shown in Fig. 4, where diffraction patterns from all 90 angles were used. As a result, the seed inputs to the multi-slice ptychographic reconstructions are much further from the ground truth, and the resulting multi-slice reconstructions are consequently worse. Nevertheless, we proceeded with the tomography step, using the 30 projections obtained from the multi-slice reconstructions and generating 60 further projections by tilting the view angle of each reconstruction by −2° and 2° as described above. These 90 projections were inputted to the modified FBP algorithm and the resulting tomographic reconstruction is illustrated in Fig. 8c. Clearly, they are not as good as the reconstructions from the complete dataset (see Fig. 6). To improve upon this, we ‘boot-strapped’ the reconstruction, re-generating the initial slice guesses using the tomographic reconstruction in Fig. 8c and re-running the multi-slice reconstructions. Repeating the angle extension method and the FBP process gave the final tomographic reconstruction shown in Fig. 8d. The improvement is obvious compared to the results shown in Fig. 8c. Importantly for future work, this demonstrates that ptychographic reconstruction can be improved by making use of the redundancy in the angular measurements^33^, since this new reconstruction is comparable to that using the full dataset of 90 angular measurements (see Fig. 6). For comparison, we also performed tomographic reconstruction using only the orthogonal projections of the selected 30 angles, as shown in Fig. 8e, in which typical radial artefacts caused by a lack of angular measurements can clearly be seen.

**Figure 9:**
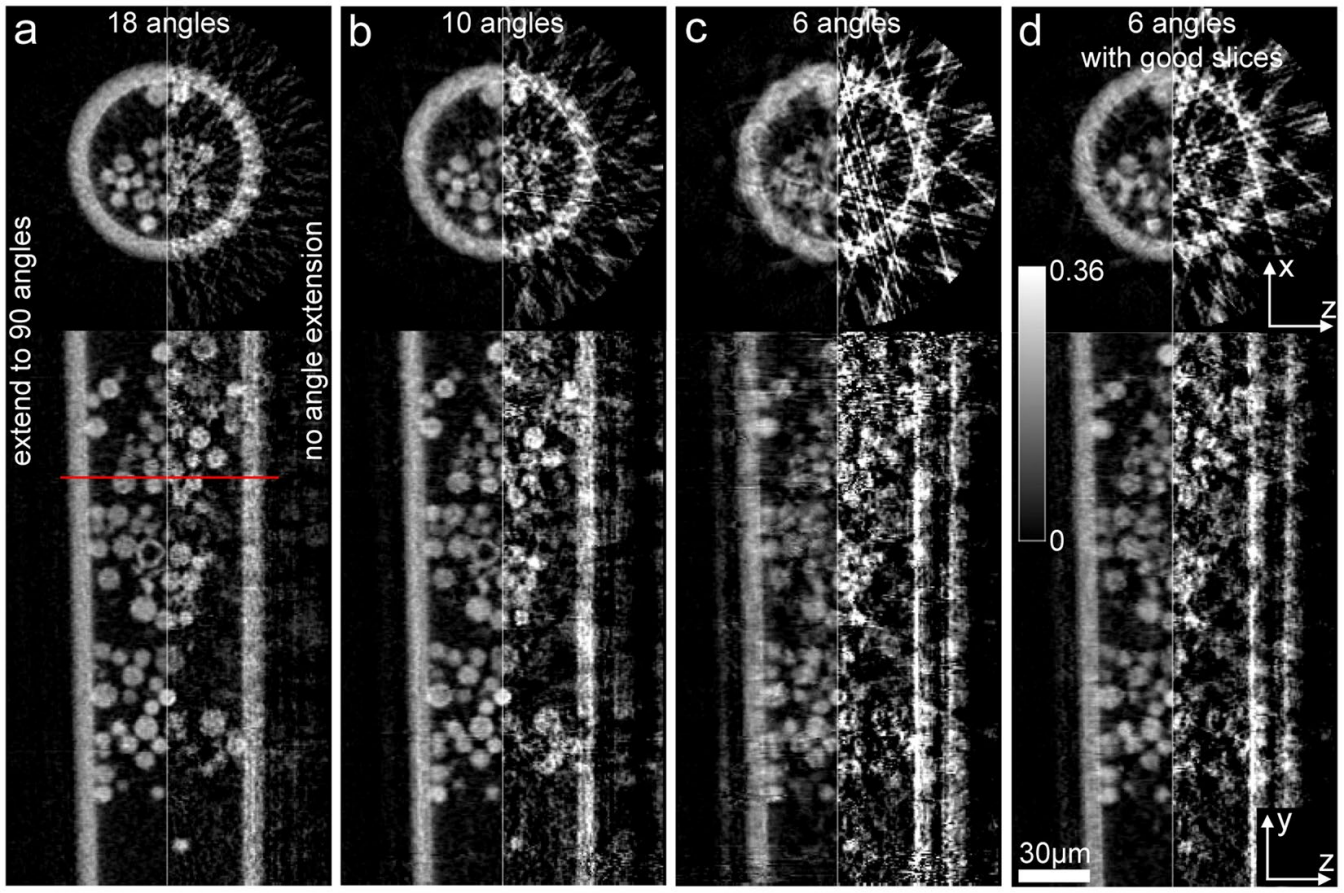
The reconstructions of multi-slice ptychographic tomography using (**a**) 18, (**b**) 10 and (**c**,**d**) 6 angular measurements. For (**d**), good slices reconstructions from the case of complete 90 angles are used for the angle extension here. The top row is the x-z cross-sections of the 3D sample reconstruction at the positions indicated by the red line. The bottom row is the central y-z cross-sections. In each pane, the left hand side is the reconstruction after the angle extension and the right hand side is the reconstruction without the angle extension.

To further push MSPT, we tried even less angular measurements: 18 (picking the middle angle in every 5), 10 (picking the middle angle in every 9) and 6 (picking the middle angle in every 15). Following the procedure detailed above, each limited-angle subset of the data was extended to 90 angles. For the 18-angle case, this involved generating four further angular projections by tilting 4° in both directions with a step size of 2°, and similarly for the 10-angle and 6-angle cases. Tomographic reconstructions using the 90 projections (with angle extension) together with their tomographic reconstructions using only the orthogonal projections (without angle extension) are shown in Fig. 9. Without the angle extension (right hand side in each panel), the reconstruction quality degrades due to the insufficient angular measurements. Most of beads are not well reconstructed when only 10 angles are used, whilst the beads are completely overwhelmed by artefacts when only 6 angles are used. However, after angle extension (left hand side in each panel), the reconstruction quality increases significantly. Although the quality drops when less angles are used, the reconstructions are still acceptable for the cases of 18 and 10 angles: all the features appear well reconstructed, but slightly noisier compared to the cases of 30 angles (see Fig. 8d) and 90 angles (see Fig. 6). Clearly, the reconstruction from the case of 6 angles (Fig. 9c) is not satisfactory, but the improvement brought by the angle extension is still very impressive. We have verified, however, that the failure in this case is mainly attributed to the poor multi-slice reconstructions, not the angle extension method, because the angle extension result (Fig. 9d) is much better when using 6 angles extracted from the “good” multi-slice reconstructions seeded with the SSPT reconstruction using all 90 angles. The poor multi-slice reconstruction in this case is caused by the poor quality of the initial guesses that can be obtained via SSPT from the very limited set of angles; we surmise that the individual ptychographic datasets do not have enough redundancy to allow the algorithm to converge accurately without good initial guesses.

## Conclusions

In this paper, we have demonstrated experimentally that the combination of multi-slice ptychography and tomography (MSPT) is able to extend the depth of field and image a 3D sample that is beyond the ability of SSPT, where single-slice ptychography is used to reconstruct the projections needed for the tomographic reconstruction. Multi-slice ptychography improves the reconstructions by accounting for propagation effects inside the 3D sample, and our experimental results have shown that MSPT is able to image a 3D sample with a thickness of 90 µm in a situation where SSPT is limited to a depth of field of approximately 26.9 µm.

Moreover, we have also demonstrated experimentally that MSPT can reduce the number of angular measurements for the tomographic reconstruction. For a particular angular orientation of the sample, its multi-slice reconstruction can be used to generate projections of a range of angles, such that projections covering 180° can be obtained without a dense set of sample rotations. Our experimental results have shown that acceptable tomographic reconstruction can be obtained using only 10 angles, which is about one seventh of the usual Crowther Limit. Even with 6 angles, the 3D reconstruction is still reasonably good, although the errors are visually obvious.

To generate multiple sample projections from a single multi-slice reconstruction, the slices are offset from each other by an amount determined by the tilted view angle. A discrepancy between the generated projection and the ground truth projection is inevitable here, because the sample sections represented by the reconstructed slices are not infinitesimally thin. However, this effect is negligible when the view angle and the section thickness are small. To further reduce the number of angular measurements, a larger view angle is required to generate sample projections, so the sections need to be thinner. An easy way to realize this is by increasing the numerical aperture (or angular range) of the illumination^22^. In other words, an illumination with a larger angular range would further reduce the minimum number of angular measurements.

Our experimental results also demonstrate that multi-slice ptychographic reconstruction can benefit from good initial guesses generated from the tomographic reconstruction, in a boot-strapping approach. The fact that the multi-slice reconstruction can be improved in this way implies that the ptychographic dataset is somewhat short of information content; in fact, a raster scan with 3 × 15 positions is indeed a rather small scan even for single-slice ptychography. Therefore, the experimental results suggest that the requirements on the translational ptychographic scan are relaxed when combined with tomographic sample rotations^33^. This prompts the possibility of implementing 3D ptychography using only rotational scanning in the future.

## Methods

### Description of the experiment

The optical experiment was carried out using a 635 nm fibre-coupled diode laser as the source. A plastic film and a doublet lens (f = 35 mm) were inserted into the beam to provide an illuminating beam with a large range of angles and a diverse structure, which has been shown to be beneficial in previous work^19,22^. The sample was placed somewhere near but upstream of the beam focus, to give an illumination size of approximately 150 µm diameter. The sample itself constituted a glass tube filled with glass beads. The tube had inner/outer diameters of approximately 70/90 µm. The beads had diameters ranging from 4 µm to 16 µm and a few of them were hollow. Both the tube and the beads scatter strongly at visible wavelengths, due to their large refractive indices relative to air. We reduced the scattering somewhat by immersing the sample in index-matching liquid (Leica, with a refractive index of 1.518), which was held in a fixed cuvette. To allow immersion of the sample, the stages and the sample were mounted upside down.

According to the Crowther Limit^16^, for tomographic reconstruction to resolve the 4 µm beads in the 90 µm tube, the number of projections needed is given by $M=\pi D/{\rm{\Delta }}r=3.14\times 90\approx 71$. Here we actually collected 90 angular measurements by rotating the sample from 2° to 180° with a step size of 2°. For each angular orientation, the sample was scanned over a 3 × 15 raster grid of x/y positions with a 20 µm step size. The illumination patch had a diameter of approximately 180 µm, giving a 89% overlap of adjacent illuminated areas during the ptychographic scan. Diffraction patterns were recorded using a 16-bit CCD detector with 2048 × 2048 pixels on a pitch of 7.4 µm^2^. The recorded diffraction patterns were binned by a factor of 4 to minimize time spent on data collection and image reconstruction.

### Description of the rotation geometry

Because of the misalignment of the sample and the rotation axis, the camera length (i.e. the distance from the sample to the camera) changed as the sample was rotated, and as a result, the pixel size of the ptychographic reconstructions changes when a Fresnel/Fraunhofer propagator is used for the propagation between the sample and the detector. This causes problems with the tomographic combination of the projections, so a virtual plane was used in the reconstruction process to ensure a fixed pixel size of the reconstructed images at different rotation angles. The position of this virtual plane was chosen to make sure of three things: 1. That the angular spectrum propagator could be adopted for the propagation between the sample and the virtual plane, so that the pixel size was kept the same for the two propagation planes; 2. That the Fresnel/ Fraunhofer propagator could still be used between the virtual plane and the detector plane; 3. That for all the angles, the sample did not extend beyond the virtual plane in the z direction.

To obtain successful ptychographic reconstructions, we still need to know the sample positions along the z-axis at each angle, so that we know how far we must propagate to reach the detector plane. To do this, we need to calculate the sample rotation trajectory in the x-z plane. As shown in Fig. 3b, the trajectory is determined by the offsets of the sample along the x- and z-axes at the 0° and 180° angles. To obtain the z position of the sample at these two angles, we tried a series of reconstruction using different z positions for each angle, and then assumed that the position giving the lowest reconstruction error was correct. Note that this trial and error process automatically accounts for the wavelength change due to the index matching fluid in which the specimen was immersed. From the two z positions, the z-offset was calculated to be about 100 µm. From the two sample reconstructions, the x-offset was calculated to be about 540 µm. As a result, the z positions (µm) of the sample at different rotation angles can be calculated by

4 $${z}_{\theta }=r\,\sin (\alpha +\theta )+100/2+180+1750,$$

where *r* is the radius of the circular rotation trajectory of the sample, *θ* is the sample rotation angle and *α* is the offset angle of the sample from the x-y plane when the sample is not rotated.

### Data availability

The datasets generated during and analysed during the current study are available from the corresponding author on reasonable request.
